# Could Electromyographic and Pressure Related Signals Identify Differences in Abdominal Activity and Postural Control between Women with and without C-Section?

**DOI:** 10.3390/s23104878

**Published:** 2023-05-18

**Authors:** Ana Figueiredo, Maria Lopes, Ana Pereira, Andreia S. P. Sousa, Cláudia Silva, Andreia Noites

**Affiliations:** Center for Rehabilitation Research, Human Movement System (Re)habilitation Area, School of Health, Polytechnic of Porto, Rua Dr. António Bernardino de Almeida, 400, 4200-072 Porto, Portugal; anamargaridafigueiredo2000@gmail.com (A.F.); mariavieiralopes@hotmail.com (M.L.); 10180119@ess.ipp.pt (A.P.); ccs@ess.ipp.pt (C.S.); arn@ess.ipp.pt (A.N.)

**Keywords:** electromyography, stabilometry, postural control, adherimetry, orthostatic position, sensors

## Abstract

Background: Scars interfere with the motor system; however, the influence of c-section scars has not been described yet. The aim of this study is to relate the presence of abdominal scars from a caesarean section with changes in postural control—stability and orientation and abdominal and lumbar neuromuscular control in the orthostatic position. Methods: Cross-sectional analytical observational study comparing healthy primiparous women with caesarean delivery (*n* = 9) and physiologic delivery (*n* = 12) who have delivered more than one year before. The relative electromyographic activity of the rectus abdominis, transverse abdominis/oblique internus and lumbar multifidus muscles, antagonist co-activation, the ellipse area, amplitude, displacement, velocity, standard deviation, and spectral power of the centre of pressure, and thoracic and lumbar curvatures, were evaluated in the standing position in both groups, through an electromyographic system, a pressure platform and spinal mouse system. In the “caesarean delivery” group, scar mobility was evaluated using a modified adheremeter. Results: Significant differences in CoP medial-lateral velocity and mean velocity were observed between groups (*p* < 0.050), while no significant differences were in the level of muscle activity, antagonist co-activation, and thoracic and lumbar curvatures (*p* > 0.05). Conclusions: The information provided by the pressure signal seems to identify postural impairments in women with c-sections.

## 1. Introduction

Pregnancy produces a variety of anatomical and physiological changes in the maternal organism to prepare for maternal-foetal organic needs and childbirth [1,2]. In the lumbar-pelvic complex, changes are noted in joints related configurations [2] and in the tone and length-tension ratio of the muscles of the abdominal core due to hormonal action and the growth of the foetus in the uterine cavity [3].

Caesarean section is a foetal delivery method carried out through a transverse incision, which covers all existing tissue until reaching the uterine wall [4,5,6,7]. It is the most used approach in Portugal since it is associated with fewer complications [8,9]. After tissue injury, it begins the cicatrisation process, which culminates with the formation of an abdominal scar [10]. Since the scars interfere with the movement, thus conditioning the functioning of the motor system, one may suspect that the presence of a scar in the abdomen will condition the role of the abdominal muscles as a stabiliser of the torso and pelvis and its interaction with the remaining muscles of the abdominal core and myofascial chains [11,12]. The potential change in muscle fibre orientation and subsequent decrease in the intensity of the electromyographic signal (mainly of the transverse abdominis, lumbar multifidus, and rectus abdominis muscles) impacts the organisation of stability, mobility, and orientation of the torso and pelvis—postural control and it may result in an increase in the variation of the centre of pressure (CoP) and changes in the physiological curves of the spine, in the orthostatic position, thus culminating in limitations concerning daily life and physical activities, as well as lumbar-pelvic pain [11,13,14,15].

The increase in the use of this surgical procedure and its consequences on women’s quality of life, along with the scarcity of articles and scientific publications related to the issue of the caesarean section, make it pertinent to assess what the influence of this scar on the system of human movement is, thus enabling a more complete and objective physiotherapy intervention in post-partum recovery.

The present study aims to assess the influence of the presence of abdominal scars resulting from a caesarean section on abdominal muscle activity and postural stability and orientation. For this, participants with and without abdominal scars resulting from caesarean section will be compared in terms of centre of pressure displacement variables (postural stability), thoracic and abdominal curvatures [16] (postural orientation), and of the abdominal and lumbar neuromuscular control—the intensity of the electromyographic signal and the antagonist co-activation index [17].

## 2. Methodology

### 2.1. Study Design

A cross-sectional design was used based on STROBE guidelines.

### 2.2. Sample

The target population of this study included Portuguese primiparous women who had delivered more than a year ago. The sample recruitment period was from 28 April to 9 June 2022. Participants who presented one or more of the following criteria were excluded: musculoskeletal and neurological conditions that may influence the activation of the abdominal core, history of persistent lumbar pain prior to and subsequent to pregnancy, vertical caesarean section scars, and smoking habits. These last two criteria are related to a higher probability of developing pathological scars. Nicotine is a vasoconstrictor substance that decreases the proliferation of erythrocytes, macrophages, and fibroblasts, compromising the efficiency of the healing process [10]. One of the most important causes of pathological healing, such as hypertrophic scars, is incorrect incision planning. An incision placed in the direction perpendicular to Langer’s lines causes unnecessary tension on the skin, pushing the wound edges apart [18], substantiating the last criterion mentioned. Only women who consented to be contacted were evaluated by resorting to other instruments, from which those who presented diastasis of the rectus abdominis greater than two fingers of the subject, types V and VI phototypes (Fitzpatrick Scale), obesity (Body Mass Index (BMI) greater than 29.99 kg/m^2^), and were undergoing menopause, were excluded.

The final sample consisted of 21 participants, divided into two groups: physiological childbirth (*n* = 12) and caesarean childbirth (*n* = 9). Data collection was carried out at the Rehabilitation Research Centre (CIR) of the School of Health of the Polytechnic Institute of Porto, and in the municipalities of Vouzela (Viseu) and Esposende (Braga), between 25 May and 14 June 2022.

### 2.3. Instruments

#### 2.3.1. Selection and Characterisation of the Sample

The TANITA scale, model BC-543 Inner Scan TM (Monitoring Your Health, Amsterdam, The Netherlands), was used to assess total body mass, fat mass, lean mass, and bone mass [19]. Its dimensions are 30 × 30 × 3 cm^3^, accounting for a mass of 2.52 kg. It has a maximum capacity of 150 kg and an accuracy of 0.1 kg per kg.

The measuring tape of COMED^®^ (COMED SAS, Strasbourg, France) has inelastic and flexible characteristics. It was used to measure the height of the participants, being 200 cm in length and bearing graduation every 1 mm [20].

The modified adheremeter, printed in a 4.6 cm-radius acetate, allowed the evaluation of the extensibility of the scar tissue in 4 directions (upper, lower, left, and right). The modified adheremeter is an adaptation of the adheremeter described in previous studies [21]. The values of the Index of Adherence Severity (AS) vary between 0 and 1: 0, representing scar immobility, and 1 represents the mobility of the completely intact scar [22].

The application of the sociodemographic and clinical questionnaire via Google Forms allowed us to collect data to characterise the population and the criteria required for participation in the study. This questionnaire includes topics related to demographic data, general health, pregnancy, and Physiotherapy intervention. In addition, it features a section for the participant to give their consent to be contacted to carry out the physical assessment face-to-face, should the participant meet the inclusion criteria.

The Fitzpatrick Scale allowed the characterisation of the phototypes of each subject through an analysis of the skin’s ability to protect or burn as a result of exposure to ultraviolet rays [23]. Because this is the only means of classification of skin phototypes, it is not possible to establish a correlation with other similar instruments. In addition, there is still no validation for the Portuguese population.

The International Physical Activity Questionnaire (IPAQ) was used to understand and obtain answers related to sedentary activity and physical activity, health-promoting, in different spheres of life. This version was validated for the Portuguese population [24], together with the coordinating group in Portugal, Mota and Sardinha. The questionnaire features a value referred to in the criterion validity bibliography with the accelerometer of *r* = 0.49 and a Cronbach α of 0.96 [24].

The Edinburgh Postnatal Depression Scale (EPDS) is a questionnaire composed of 10 items, which allowed the evaluation of the presence and intensity of symptoms of depression in the previous 7 days, using a Likert-type scale of 0 to 3 [25]. This scale is validated for the Portuguese population [26].

#### 2.3.2. Data Acquisition

Surface electromyography was used to evaluate the muscle activity of rectus abdominis (RA), transverse abdominis (TrA/OI), and lumbar multifidus (MF) bilaterally. The bioPLUXc Research (Plux Ltd., Lisbon, Portugal) wireless signal acquisition system was used. The signals were collected with a sampling frequency of 1000 Hz, pre-amplified on each electrode through a differential amplifier with an adjustable gain setting (25–500 Hz; common mode rejection rate (CMRR): 110 dB at 50 Hz, input impedance of 100 MΩ, and gain of 1000). Self-adhesive and disposable silver chloride electromyography electrodes (EMG), with a characteristic of 3.4 cm^2^ × 3.6 cm^2^ and 1 cm in diameter of the conductive circular area, were used. These were placed in bipolar configuration with centres of the detection surfaces 2 cm apart [27]. The reference electrode features the same physical characteristics except for its dimensions, which were 3.5 cm^2^ × 4.2 cm^2^ and 1 cm in diameter of the conductive circular area. This electrode was placed at the level of the spinous apophysis of vertebra C7. The sensors were connected via Bluetooth to a computer. The MonitorPlux software Version 2.0 was used to display and collect the electromyographic signal.

The pressure platform PhysioSensing (Sensing Future Technologies, Coimbra, Portugal) was used to evaluate the variation of CoP displacement. It consists of a rigid surface with pressure sensors, sized 61 cm^2^ × 58 cm^2^, 1cm thickness, 4 kg of mass, 40 cm^2^ × 40 cm^2^ active surface, 1600 resistive-type sensors, sized 1 cm^2^ × 1 cm^2^, maximum pressure in each sensor of 100 N/cm^2^ and frequency of 100 Hz. This pressure platform allows the evaluation of postural stability by describing its components objectively and quantitatively. The 19.0.1.0 software was used, and the database was exported in PDF format [28].

The non-invasive measuring device Spinal Mouse, IDIAG M360 (Idiag AG, Zurich, Switzerland), allowed the evaluation of the angle and shape of the thoracic and lumbar spine in the frontal and sagittal planes. The equipment features two rotating wheels accompanying the spinous apophyses of the spine while measuring the distances and angles between the vertebrae, which are later transferred to a computer. Data are transferred every 1.3 mm as the instrument slides along the spine, thus providing a sampling frequency of approximately 150 Hz. This information is then used to calculate the relative positions of each vertebra, the angles between the vertebrae, and the total angle of the frontal and sagittal curves [29].

### 2.4. Procedures

#### 2.4.1. Selection and Characterisation of the Sample

The selection of the sample was initially carried out through the application of a Google Forms questionnaire. After completing the questionnaire, the participants who met the inclusion criteria and accepted further contact for a face-to-face physical assessment were contacted.

Within the face-to-face context, the anthropometric evaluation was performed on each participant regarding height, body mass, and BMI, thereby excluding those who presented a degree of obesity. To measure the subject’s height, the participant positioned themselves barefoot while keeping their feet at the width of the hips, with heels, buttocks, shoulder blades, and occipital against a measuring tape glued to the wall [30]. To obtain data on body composition—total body mass (kg), fat mass (%), lean mass (kg), and mineral bone mass (kg)—the participants maintained the orthostatic position on the scale, with bare feet and their upper limbs along the body, facing forward [31]. The Fitzpatrick scale was then handed to the participants to characterise their individual phototypes. Type V and VI phototypes were excluded since these present a greater tendency to develop fibroproliferative scars [32].

The evaluation of the abdominal diastasis was performed by palpation of the distance between the medial edges of the RA. This evaluation was carried out in dorsal decubitus with knees bent at 90°, feet supported on the observation table, and upper limbs alongside the torso, comparing the distance at rest and during the abdominal crunch (after 3 to 5 s of contraction at the end of exhalation) [33,34]. The measurement occurred at the origin of the xiphoid apophysis, 3 cm above the navel and 2 cm below the navel, with only one measurement for each point [35]. All subjects with a distance between RA greater than 2 fingers of the subject were deemed to have diastasis and, for this same reason, were excluded from the study [36].

To identify the existence of depressive symptoms in the post-partum period and the level of physical activity in the pre, during, and post-pregancy, participants were asked to complete the EPDS and IPAQ, respectively.

The modified adheremeter was used to evaluate the mobility and extensibility of the scar [21] only in the “caesarean delivery” group (CDG). Three marks were created in the scar (points 0, 1, and 2), whose extensibility was evaluated in the 4 directions (upper, lower, left, and right) in millimetres. The extensibility of the tissue was also measured, on the right side, 5 cm from the navel.

#### 2.4.2. Data Acquisition

The skin of each participant was properly prepared for the electromyographic evaluation. Hair removal and exfoliation of the area with Nuprep were performed to remove dead skin cells from the skin surface. Subsequently, it was cleaned with ethyl alcohol (70%) to remove oil and dead cells. Through this preparation, the skin impedance levels were reduced, thus ensuring a better acquisition of the electromyographic signal [37]. After 5 min of skin preparation, the self-adhesive electrodes were placed. They were applied parallel to the orientation of muscle fibres, according to the references described in Table 1 [38,39], with confirmation of the site through palpation during muscle contraction. The cables associated with the electrode were taped to the subject to ensure their proper positioning and reduce the signal noise associated with the movement of the cables [37]. The reference electrode was placed on the spinous apophysis of vertebra C7. The quality of the electromyographic signal was analysed to control the existence of electrical interference and/or crossing of signals through the analysis of the baseline and the frequency spectrum [37].

For the normalisation of the electromyographic signal, reference contractions were used. The McGill flexors test was used for the abdominal muscles (RA and TRA), whereas the Sorensen test [40] was used for the MF. In both tests, 3 isometric 5-s contractions were requested, with a 5-s rest interval [40]. The description of the tests is shown in Table 2. This procedure was carried out at the end of the evaluation of the postural set under study to minimise the onset of muscle fatigue during the evaluation.

The processing of the electromyographic signal was performed using Acknowledge software (Version 3.9). A Butterworth 2nd Order band filter, between 20 Hz and 500 Hz, was applied to remove any electrical noise affecting the signals obtained in the standing position and in the McGill and Sorensen tests. In cases of cardiac signal detection, a Butterworth 2nd Order band filter, between 50 Hz and 500 Hz, was used [41]. Subsequently, the Root Mean Square (RMS) was calculated in a sliding window of 100 samples. The mean value of the central 60 s was considered for the standing position. The maximum peak mean of the 3 repetitions was considered a reference value [41] for the normalisation tests.

The mean value of the electromyographic signal obtained for each muscle was used in the analysis, normalised by the signal obtained in the Sorensen tests for the MF and McGill’s flexors for the RA and TrA/OI through the following equation:

$$\mathrm{Relative}\;\mathrm{Intensity}\;\mathrm{of}\;\mathrm{Muscle}\;\mathrm{Activation}=\frac{\mathrm{Average}\;\mathrm{Intensity}\;\mathrm{in}\;\mathrm{the}\;\mathrm{Standing}\;\mathrm{Position}}{\mathrm{Reference}\;\mathrm{Value}}\;.$$

In addition, the co-activation value between agonist (TrA) and antagonist (MF) was calculated using the equation below [42]:

$$\mathrm{Relative}\;\mathrm{Value}\;\mathrm{of}\;\mathrm{Antagonist}\;\mathrm{Co}-\mathrm{activation}=\frac{\mathrm{Relative}\;\mathrm{Antagonist}\;\mathrm{Intensity}}{\mathrm{Relative}\;\mathrm{intensity}\;\mathrm{of}\;\mathrm{agonist}+\mathrm{antagonist}}\;.$$

**Figure 1:**
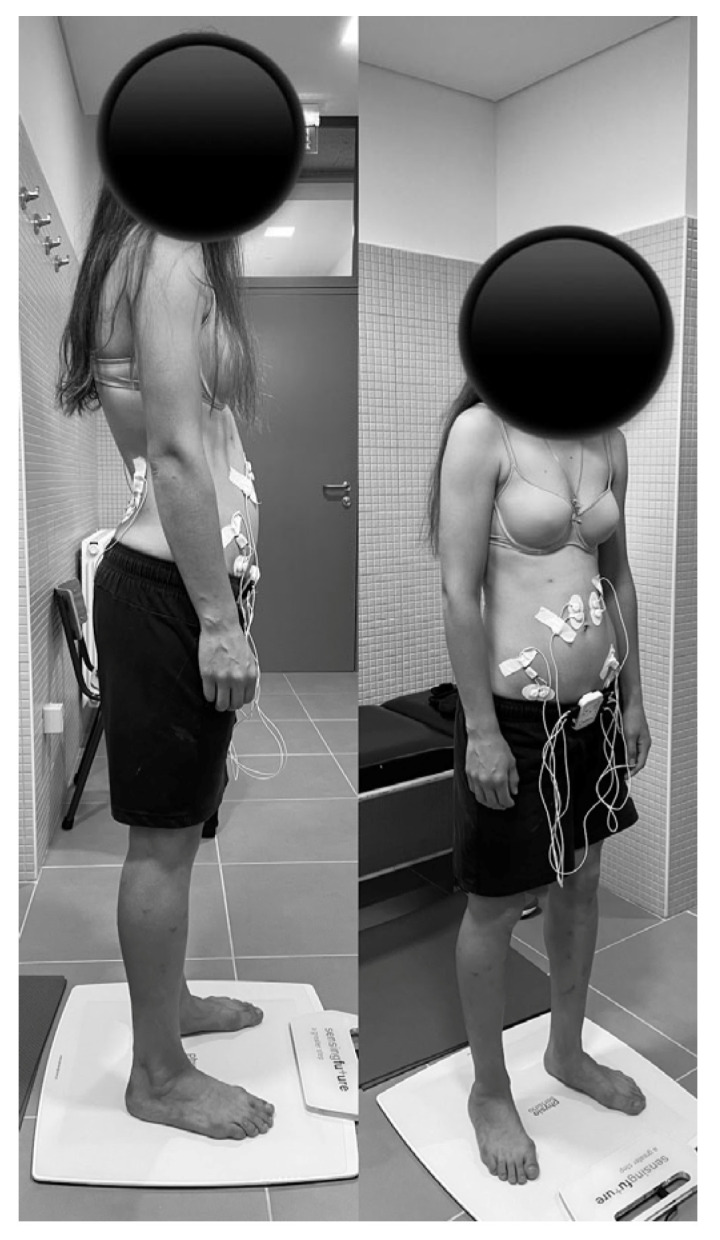
Positioning of the participant with caesarean section scar during the evaluation of the orthostatic position.

The collection of the electromyographic signal from the RA, TrA/OI, and MF muscles was carried out simultaneously with the collection of data from the pressure platform (Figure 1).

The pressure platform allowed us to evaluate the displacement of the COP in the orthostatic position. Participants were asked to position themselves barefoot, according to the references provided by the platform protocol, with their upper limbs along the body while keeping their gaze fixed on the achromatic target positioned 2 m away at eye height [43]. The “Body Sway” protocol was used, in which the participant maintained the test position for 60 s, with a resting period of 120 s, between the 3 repetitions [44].

Finally, in both groups, the physiological curves of the spine were evaluated using the Spinal Mouse instrument. The participants were evaluated in the standing position, barefoot, with a comfortable support base, their upper limbs along the body, and naked torso. The evaluator marked the references for C7 and S3 and then moved along the spine between these segments with the equipment, making 3 measurements for each participant.

After the collection and processing of the data provided by the instruments, they were exported to an Excel spreadsheet.

#### 2.4.3. Statistical Analysis

For the statistical analysis, the software Predictive Analytics Software Statistics 21 (SPSS^®^ IBM Corporation, Chicago, IL, USA) was used, with a significance level of 0.05 and a confidence interval of 95%.

Since the size of the CDG was less than 10, the test could not be applied to normality, due to the fact that the distribution of normality in the variables could not be assured. Thus, the Mann-Whitney test was used in two independent samples to analyse quantitative data (age, height, body mass, BMI, level of muscle activity, stabilometry, amplitude of vertebral column curvature). Regarding the qualitative data (IPAQ, “Consult with a Physiotherapist”, EPDS, Fitzpatrick Scale), hypothesis tests were carried out for proportions of 2 independent samples (Chi-Square Tests). As the variables do not follow a normal distribution, the median, the 25 and 75 percentiles, and the percentage values were used for the descriptive analysis.

The estimate of the sample size was calculated based on the following equation:

$$n=\left(\frac{Z_{\alpha /2}{}^{2}\;p\;\left(1-p\right)}{E^{2}}\right)$$

- *n*: sample size;
- *p*: expected proportion (*p* = 0.5);
- *Z*: normal distribution value for a specific confidence level (*Z* = 1.96);
- *E*: confidence interval size (*E* = 0.05).

This resulted in a recommended value of 385 participants. However, given that, for this study, it was not possible to recruit the desired number of participants, statistical power was calculated for the sample considered. For such, the G*Power version 3.1.9.6 (Kiel University, Kiel, Germany) was used a posteriori. A significance level of 0.05 and 2 tails were considered.

## 3. Results

### 3.1. Sociodemographic and Clinical Characterisation

**Figure 2:**
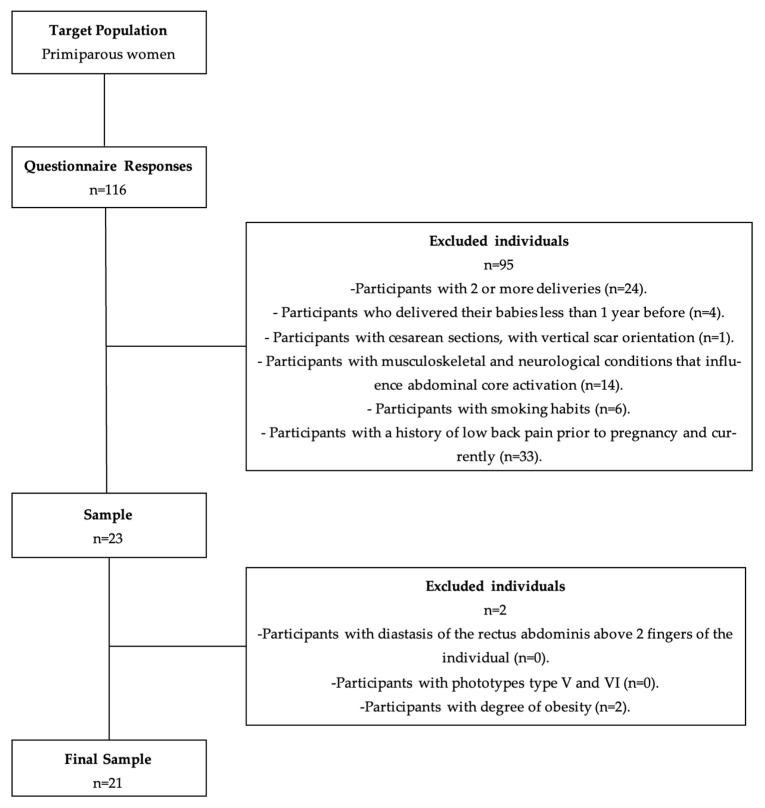
Sample constitution diagram.

As a result of the questionnaire distribution, 116 responses were obtained, of which 95 were excluded. Of the remaining 23 participants, 2 were excluded at the time of the physical evaluation. Thus, the final sample consisted of 21 women. The exclusion criteria are described in Figure 2.

After analysing the quantitative variables age, height, body mass, and BMI, there were no statistically significant differences between groups (*p* > 0.05) (Table 3). The median values (P25; P75) of each parameter used in characterising the population are described in Table 3.

There were also no statistically significant differences between the groups in the variables phototype, EPDS, IPAQ, “time after childbirth”, and “consult with a physiotherapist” before and after pregnancy (*p* > 0.05) (Table 4). The percentage values (%) of each parameter used to characterise the population are described in Table 4.

Regarding the evaluation of scar mobility in the CDG, evaluated according to the modified adheremeter, AS values between 0.22 and 0.79 were found, with a median of 0.55 (0.47; 0.67).

### 3.2. Level of Muscle Activity

After analysing Table 5, it is possible to observe a trend for bilateral activation of the upper RA muscles in the CDG and the opposite trend for the remaining muscles. However, no significant statistical differences were observed in all muscles tested, as well as in co-activation levels, between the two groups (*p* > 0.050).

### 3.3. Postural Control: Stability and Orientation

**Figure 3:**
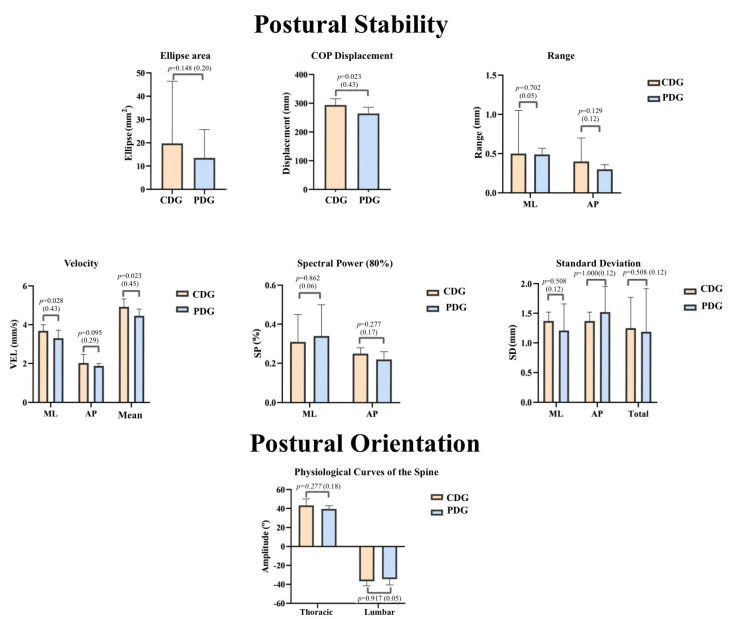
Values obtained in the different components of postural stability and orientation in the medial-lateral (ML) and anteroposterior (AP) direction in the “caesarean delivery” groups (CDG) and “physiological delivery” (PDG). The median values (P25; P75), the *p* values of the Mann-Whitney test, and the respective statistical power are presented.

From the analysis of Figure 3, we can observe a trend for higher COP displacement, ellipse, velocity (VEL), and standard deviation (SD) values in the CDG, and significant differences were observed in the parameters COP displacement, medial-lateral velocity (ML VEL), and mean velocity (Mean VEL) (*p* < 0.050).

In relation to the data regarding postural orientation, we observed values that tend to be higher in the caesarean section group, both at the thoracic and lumbar curvature levels. However, there were no significant statistical differences between the two groups (*p* > 0.050). Regarding the statistical power, it was reduced in both curves under analysis (Figure 3).

## 4. Discussion

This study aimed to analyse the influence of caesarean scars in postural control, namely at the level of abdominal muscle activity, torso orientation, and variables related to COP displacement.

Regarding the level of the intensity of the electromyographic signal, divergent trends were identified between groups, although with no significant differences. There was a higher intensity in superficial muscle activation (RA) and lower deep muscle activity (TrA/OI and ML) in the CDG. The TrA/OI and MF muscles are considered deep muscles and integral parts of the local muscle system, responsible for controlling the stability of the intervertebral joints of the spinal segments [15,45], and for generating a positive intra-abdominal pressure, thus allowing an automatic and anticipatory stabilising response [12]. A balanced but also adaptive co-activation is crucial for greater support and postural control [12]. On the other hand, the RA is part of the global muscle system. This system consists of torque-generating muscles, which are more dependent on the task being performed since they control the orientation of the spine and balance the external loads resulting from the spatial relationship between the thorax, pelvis, and spine [12,15,45].

The level of muscle activity and the interaction between the aforementioned systems influence posture control [12,45]. Any imbalance in this interaction may place certain muscles in overload, thus conditioning the functionality of the lumbar-pelvic complex and may affect the subjects’ quality of life [45]. The Caesarean section scar may initially compromise the function of the abdominal muscles since the accumulation of new scar tissue (with characteristics different from those presented by the original tissue) is associated with reduced organ performance [10]. The results reflect the previously described hypothesis; however, not in its entirety, since women featuring a caesarean section scar display lower levels of abdominal muscle activity only in what the deep muscles are concerned. Regarding the antagonist co-activation variable, no differences were found between the groups.

Regarding the data obtained in the postural stability component, in the CDG, a trend was observed, which translated into higher values of displacement, ellipse, VEL, and SD of the COP, when compared to the PDG, with significant differences in the parameters displacement, ML VEL and Mean VEL of the COP. These results suggest that the CDG presents lower postural stability and a less efficient postural control system since the values of AD, displacement, and ellipse area parameters were higher and less effective since the COP SD values were also higher [41]. The EP and CoP VEL parameters allow us to infer the degree of control that the CNS has to exercise over the CoP so that it is possible to maintain the desired postural set [46]. This trend to decrease stability in the CDG may be closely related to the presence of the abdominal scar due to the factors explained above.

As to the postural orientation parameter, it was not possible to demonstrate any trend or significant differences between groups, regarding lumbar and thoracic curves, possibly due to the variety of factors that influence postural orientation that were not subject to control. The automatic postural responses are shaped by sensory characteristics and CNS mechanisms related to factors such as expectations, attention, sensory-motor experience, environmental context, and intention, as well as pre-programmed muscle activation patterns called synergies [17].

Some limitations were identified in this study. The collection of the electromyographic signal is subject to several factors that altered the output, such as the subject’s body composition, the characteristics of the tissue, the physiological “cross-talk” phenomenon, potential changes in the geometry between the muscle belly and the electrode, external noise and the quality of the electrodes and associated amplifiers [47]. During the collection, a random error was detected in acquiring the electromyographic signal of the LRA muscle. For this reason, the electromyographic values of this muscle were devalued and excluded from the analysis. A limitation of the pressure platform is the use of a standardised position of the feet, which requires all subjects to adopt the same positioning, which is only validated for the Nordic population, not taking into account the characteristics of each subject, thus not allowing the acquisition of the typical orthostatic position [16].

It is important to mention that the lack of differences between groups may be justified because the task under analysis is rather undemanding since it may not be challenging enough to significantly recruit the selected musculature. Nevertheless, it should also be noted that the orthostatic position is unstable, thus requiring constant adjustments in the neuromuscular system to maintain the centre of gravity within the support base [16], corroborated by the statistically significant difference detected between groups and their stabilometric parameters.

The scarcity of literature addressing the topics inherent to this research was considered an obstacle to the rationale of this study. However, this absence emphasises its innovative nature. The fact that the sample was recruited voluntarily, and, after such recruitment, some subjects withdrew their participation, led to the existence of selection bias, resulting in a small sample size while hindering the representativeness of the population and decreasing the statistical power, thus compromising the external validity of this study. However, this study can represent a starting point for further research into this matter. Regarding the questionnaires used to characterise the sample, all were subject to memory bias since the questions referred to past events.

For future studies, we suggest the recruitment of a larger sample together with the collection of data related to gestational time and body mass of the baby at birth. It would also be relevant to use more stringent inclusion and exclusion criteria to control confounding variables and allow data reproducibility. At the same time, it would be interesting to evaluate other postural sets or functional tasks to verify whether the complexity of the task changes the results. It would also be important to evaluate the remaining muscles of the abdominal core, such as the diaphragm and the pelvic floor, due to their functional relationship. Investigating the changes resulting from pregnancy may also be a valuable complement to this study by adding a group of nulliparous subjects to the study, as well as creating subgroups within the physiological birth group (with and without the use of instrumentation and episiotomy). It is important to note that the instruments used only allowed us to evaluate some of the components that define postural control.

## 5. Conclusions

In the CDG, lower values were found in the ML VEL and Mean VEL of the CoP, thus indicating lower postural stability when compared to the PDG. There were no significant differences in the variables of the level of muscle activity and postural orientation.

The information provided by pressure sensors seems to identify postural changes in women with caesarean section scars. Further studies on this topic are required.

## Figures and Tables

**Table 1:** Location of the electrodes of the rectus abdominis (RA), transverse abdominis (TrA/OI), and lumbar multifidus (MF) muscles.

Muscle	Anatomical References
RA	3 cm above the navel and 2 cm to the side of the midline [39]
TrA/OI	2 cm medial and below the anterior superior iliac spines [39]
MF	Crossing point between the line connecting the posterior superior iliac spines and the L1-L2 interspace, and the point at 2 or 3 cm to the side of the spinous apophysis of L5 [38]

**Table 2:** Description of the tests used for the normalisation of the electromyographic signal.

Test	Muscles to Evaluate	Positioning	Procedure
Sorensen test	Lumbar Multifidus	The participants positioned themselves supine, with the lower part of the body manually fixed to the observation table, with the anterior-superior iliac spines aligned with the edge of the observation table and their hands resting on the opposite shoulders. The participants maintained an upright spine position during the test [40].	5 s of contraction 5 s of rest between each contraction 3 repetitions in total
McGill flexors test	Transverse Abdominis/Oblique Internus Rectus Abdominis	The participants positioned themselves seated with the torso flexed at 60°. Both knees were flexed at 90°. The hands rested on opposite shoulders. The participant maintained the flexion of the torso during the test [40].	

**Table 3:** Anthropometric characterisation of age, height, body mass, and BMI (Body Mass Index). Featured are the descriptive values of the Median (P25; P75) and the *p* values of the Mann-Whitney test.

	Groups		Differences between Groups
	Caesarean Delivery	Physiological Delivery	*p*-Value
Age (years)	36.00 (34.50; 37.00)	36.50 (31.25; 42.50)	0.702
Height (m)	1.66 (1.59; 1.72)	1.64 (1.59; 1.65)	0.464
Body Mass (kg)	60.30 (53.15; 63.45)	57.45 (55.13; 59.18)	0.754
BMI (kg/m^2^)	21.40 (20.50; 22.90)	21.70 (20.50; 22.63)	1.000

**Table 4:** Characterisation of the sample according to the Phototype, the Postnatal Depression Scale (EPDS), the International Physical Activity Questionnaire (IPAQ), and the variables “Time after childbirth”, “Consult with a physiotherapist prior to pregnancy”, and “Consult with a physiotherapist after pregnancy”. The percentage descriptive values and the *p* values of the Chi-Square test are presented below.

			Groups		Differences between Groups
			Caesarean Delivery	Physiological Delivery	*p*-Value
Phototype		Type I (%)	-	-	0.230
		Type II (%)	-	16.7	
		Type III (%)	66.7	41.7	
		Type IV (%)	33.3	41.7	
		Type V (%)	-	-	
		Type VI (%)	-	-	
EPDS		Increased risk of depression	11.1	8.3	1.000
IPAQ	Pre	Low	22.2	25.0	0.409
		Moderate	11.1	33.3	
		High	66.7	41.7	
	During	Low	33.3	25.0	0.835
		Moderate	44.4	41.7	
		High	22.2	33.3	
	After	Low	33.3	33.3	0.896
		Moderate	33.3	25.0	
		High	33.3	41.7	
“Time after childbirth.”		1 to 2 years	77.8	50.0	0.367
		More than 2	22.2	50.0	
“Consult with a Physiotherapist prior to pregnancy”.		Yes	88.9	41.7	0.067
“Consult with a Physiotherapist after pregnancy”.		Yes	33.3	33.3	1.000

**Table 5:** Intensity of electromyographic signal in the left rectus abdominis (LRA) and right rectus abdominis (RRA), left transverse abdominis/oblique internus (L TrA/OI) and right transverse abdominis/oblique internus (R TrA/OI), and left lumbar multifidus (LMF) and right lumbar multifidus (RMF) muscles. The Median descriptive values (P25; P75), the *p* values of the Mann-Whitney test, and the respective statistical power are presented below.

		Groups		Differences between Groups	
		Caesarean Delivery	Physiological Delivery	Statistical Power	*p*-Value
MUSCLE ACTIVITY	LRA	0.70 (0.10; 0.82)	0.50 (0.18; 0.72)	0.14	0.464
	RRA	0.30 (0.09; 0.46)	0.19 (0.08; 0.41)	0.06	0.754
	TrA/OI L	0.32 (0.13; 0.44)	0.33 (0.19; 0.57)	0.09	0.571
	TRA/OI R	0.24 (0.14; 0.36)	0.38 (0.20; 0.44)	0.07	0.208
	MFL	0.10 (0.08; 0.26)	0.21 (0.09; 0.25)	0.05	0.345
	MFR	0.07 (0.04; 0.10)	0.05 (0.03; 0.08)	0.11	0.422
	Co-activation	0.22 (0.16; 0.50)	0.29 (0.19; 0.39)	0.06	0.970

## Data Availability

Not applicable.
